# Systemic Analysis on the Features of Immune Microenvironment Related to Prognostic Signature in Head and Neck Squamous Cell Carcinoma

**DOI:** 10.3389/fgene.2022.860712

**Published:** 2022-05-11

**Authors:** Kaixin Su, Zekun Zhou, Qiao Yi, Junjie Liu, Tiao Luo, Xinyan Cui, Haixia Zhang

**Affiliations:** ^1^ Academician Workstation for Oral-Maxilofacial and Regenerative Medicine, Central South University, Changsha, China; ^2^ Hunan Key Laboratory of Oral Health Research, Central South University, Changsha, China; ^3^ Hunan 3D Printing Engineering Research Center of Oral Care, Central South University, Changsha, China; ^4^ Hunan Clinical Research Center of Oral Major Diseases and Oral Health, Central South University, Changsha, China; ^5^ Xiangya Stomatological Hospital, Central South University, Changsha, China; ^6^ Xiangya School of Stomatology, Central South University, Changsha, China; ^7^ The Oncology Department of Xiangya Second Hospital, Central South University, Changsha, China

**Keywords:** bioinformatics, immune microenvironment, prognosis, clinically significant groups, head and neck squamous cell carcinoma

## Abstract

**Background:** Head and neck squamous cell carcinoma’s tumor immune microenvironment (TIME) plays an important role in tumorigenesis and progression, but its clinical significance remains unclear. Therefore, the TIME needs to be better understood in order to improve the response of diagnosis and therapy.

**Methods:** The gene expression and clinical data of 569 HNSCC patients were obtained from The Cancer Genome Atlas (TCGA) and the Gene Expression Omnibus (GEO). Immune-related genes (IRGs) from the ImmPort database were used for immunotyping of HNSCC patients, and independent GEO datasets were used for subtype verification and comprehensive molecular identification.

**Results:** The patients were divided into three subtypes (C1, C2, and C3) related to different gene expression profiles. The three subtypes showed widely different patterns in tumor genetic distortion, immune cell composition, cytokine profile, and so on, verifying that the immune-enhanced C2 subtype was associated with better prognosis. In addition, the stroma-deficient C1 subtype may be more efficient for the immune response than the C3 subtype. Furthermore, using WGCNA on the IRGs of those three subtypes, we found two C2-positive gene modules closely related to infection- and immune-associated pathways in the Kyoto Encyclopedia of Genes and Genomes (KEGG) pathway database, and the two modules had 22 common pathways.

**Conclusion:** This study improves the power for prognosis prediction and develops new therapeutic strategies to stratify HNSCC patients into clinically significant groups through TIME-related prognostic signature.

## Introduction

Head and neck squamous cell carcinoma (HNSCC) is a kind of squamous cell carcinoma that occurs in the nasal cavity, lips, mouth, salivary glands, and/or throat (Ferlay et al., 2019). It ranked 6^th^ place among all common cancers worldwide, and the overall survival rate has not improved in decades (Bray et al., 2018). HNSCC morbidity varies depending on patient age, gender, geographical region, and risk factors associated with tumor progression (Okami, 2016; Szewczyk et al., 2018; Cardin et al., 2019). HNSCC currently adopts a comprehensive treatment that combines surgery, radiotherapy, chemotherapy, and target therapy. However, this approach does not come with a satisfactory outcome (Johnson et al., 2020). The cells in the tumor microenvironment (TME) contain tumor cells, immune cells, and stromal cells that play vital roles in judging tumor stage and treating HNSCC (Curry et al., 2014; Plzák et al., 2019; Wang et al., 2019).

HNSCC is a predominantly immunosuppressive and highly heterogenous tumor, which is confirmed by a multi-omics study (Bhat et al., 2021). The immune cells in the TME are involved in tumorigenesis and development, which may also trigger or decide the tumor development stage (Solomon et al., 2018). Immune escape has been identified as a cancer marker (Oliva et al., 2019). In addition, tumor-associated macrophages and myeloid-derived suppressor cells (MDSCs) within HNSCC tissues have a great influence on the immune escape mechanisms of cancer cells, and they are verified to show a marked correlation with the prognostic outcome for patients (Chen et al., 2017a; She et al., 2018). At present, the precise molecular mechanism of TIME cells in regulating HNSCC development is largely unclear. Further analysis and understanding of the TIME will contribute to a more accurate classification of patients based on their TIME and will help better observe overall survival rates and improve therapy responsiveness.

The role of the TIME in HNSCC has been studied previously, but this idea has not been considered in daily clinical practice, and this may be related to the deficient samples, insufficient evidence, and excessive data fitting in many studies. Therefore, comprehensive detection of all tumor phenotypes along with global immune profiles through high-throughput sequencing technology is necessary. In this bioinformatics analysis, we use the ImmPort immune-related genes and TCGA gene expression data and clinical information to establish HNSCC immune subtypes. Subsequently, GEO data are imposed to subtype verification and comprehensive molecular identification. This work aimed to examine the differences in TIME phenotypes, together with the related clinical implications within HNSCC.

## Materials and Methods

### HNSCC Datasets and Preprocessing

The NCBI GEO (https://www.ncbi.nlm.nih.gov/geoprofiles/) and TCGA (https://cancergenome.nih.gov/) databases were used to obtain RNA-seq data and clinical information within HNSCC. The 500 HNSCC samples (Supplementary Table S1) were obtained from the TCGA database in accordance with the criteria below: 1) follow-up data were available; 2) the gene expression data in HNSCC were accessible; and 3) genes whose expression quantities were more than 0 within every sample occupied over 30% among all genes discovered from the immune gene set (Rooney et al., 2015; Charoentong et al., 2017). 270 HNSCC samples (Supplementary Table S1) obtained *via* the GEO GSE65858 dataset were enrolled in the external validation cohort (Wichmann et al., 2015). In addition, the Illumina platform was used to analyze RNA-seq data. Moreover, single-nucleotide polymorphism (SNP), together with fragments per kilo base of gene per million fragments mapped with upper quartile normalization (FPKM-uq), was obtained *via* the TCGA Data Portal.

### Collection of Immune-Related Data and Immune Score Analysis

The scores for six immunocyte types within HNSCC were obtained *via* the Timer database (https://cistrome.shinyapps.io/timer/) (Li et al., 2017). For all samples within 13 metagenes, their scores were decided by the average log2-transformed levels of all genes in that metagene (Safonov et al., 2017). In addition, the score of each sample in the immune-related pathway was calculated using the R package GSVA according to the expression level of each associated gene for each sample in the 28 immune pathways (Bindea et al., 2013). Moreover, the R package estimate was adopted to calculate both stromal and immune scores (Becht et al., 2016).

### Determination of HNSCC Subtypes According to IRGs

The IRGs were searched using the ImmPort database (https://immport.niaid.nih.gov), and then samples were selected that had both the expression profile and the clinical follow-up for this study. Next, the immune gene set with the highest expression level was extracted from the spectrum, and the top 50% of MAD (Median Absolute Deviation) genes were further screened.

Consistent clustering was performed using the ConsensusClusterPlus package (Wilkerson and Hayes, 2010), and the molecular subtypes were screened on the basis of expression patterns of IRGs. The Kolmogorov–Smirnov test was applied to identify those highly expressed genes within certain subtypes. Multiple testing was performed using Bonferroni correction. A false discovery rate (FDR) was calculated through the Benjamini–Hochberg approach, and genes whose FDR values were less than 0.05 were identified as significantly upregulated. Thereafter, the 100 most significantly upregulated genes were screened from every subtype for principal component analysis (PCA) so as to differentiate the diverse molecular subtypes (Chen et al., 2017b).

### Analysis of the Gene Co-Expression Network

The WGCNA R package was used in determining the common pathways involved in those six gene modules (Langfelder and Horvath, 2008). In the scale-free co-expression network, the node log (k) and the connection degree k showed negative correlations with node probability log (P(k)), with the correlation coefficient of >0.8. This study converted the expression matrix to the adjacency matrix and then to the topological matrix. The topological overlap matrix (TOM, unsigned type) was used to construct the WGCNA network and detect modules. The power β was 6, the minimum size of a module was 30, and the threshold height of branch merge was 0.25. In addition, the clusterProfiler in the R package was used for KEGG pathway analysis at the threshold of FDR <0.05. Then, Cytoscape 3.7.1 was applied in visualizing genes showing significant correlation (Kohl et al., 2011).

### Verification of Three Subtypes Associated With Immune Status

For validating those three subtypes associated with immune status discovered based on the TCGA cohort, all genes within co-expression gene modules (turquoise and yellow) showing tight correlation with the C2 subtype were chosen, and associations of genes with modules were determined. In addition, those GEO cohort–derived cancer samples were divided according to those featured genes at a coefficient of correlation of >0.5. In addition, for the validation set, their gene expression data were adopted for classifying samples through the support vector machine (SVM). Moreover, to better validate those three subtypes associated with immune status, 270 samples with normalized data were obtained based on the GSE65858 dataset, followed by SVM classification.

### Statistical Methods

Associations of clinical variables with subtypes were determined through Fisher’s exact test or chi-square test. The FDR of the Benjamini–Hochberg method was obtained to correct multiple testing. Survival curves for those three immune subtypes were compared by the Kaplan–Meier method and log-rank test. A difference of *p* < 0.05 (two-tailed) indicated statistical significance. Both gene expression levels and immune scores were compared across diverse HNSCC subtypes through Student’s t-test. In multiple testing, the false-positive rate was reduced through FDR correction. Statistical analysis was conducted using R software (version 3.5.3, http://www.R-project.org).

## Results

### Construction of Three Immune Gene–Based Clusters Within HNSCC

**FIGURE 1:**
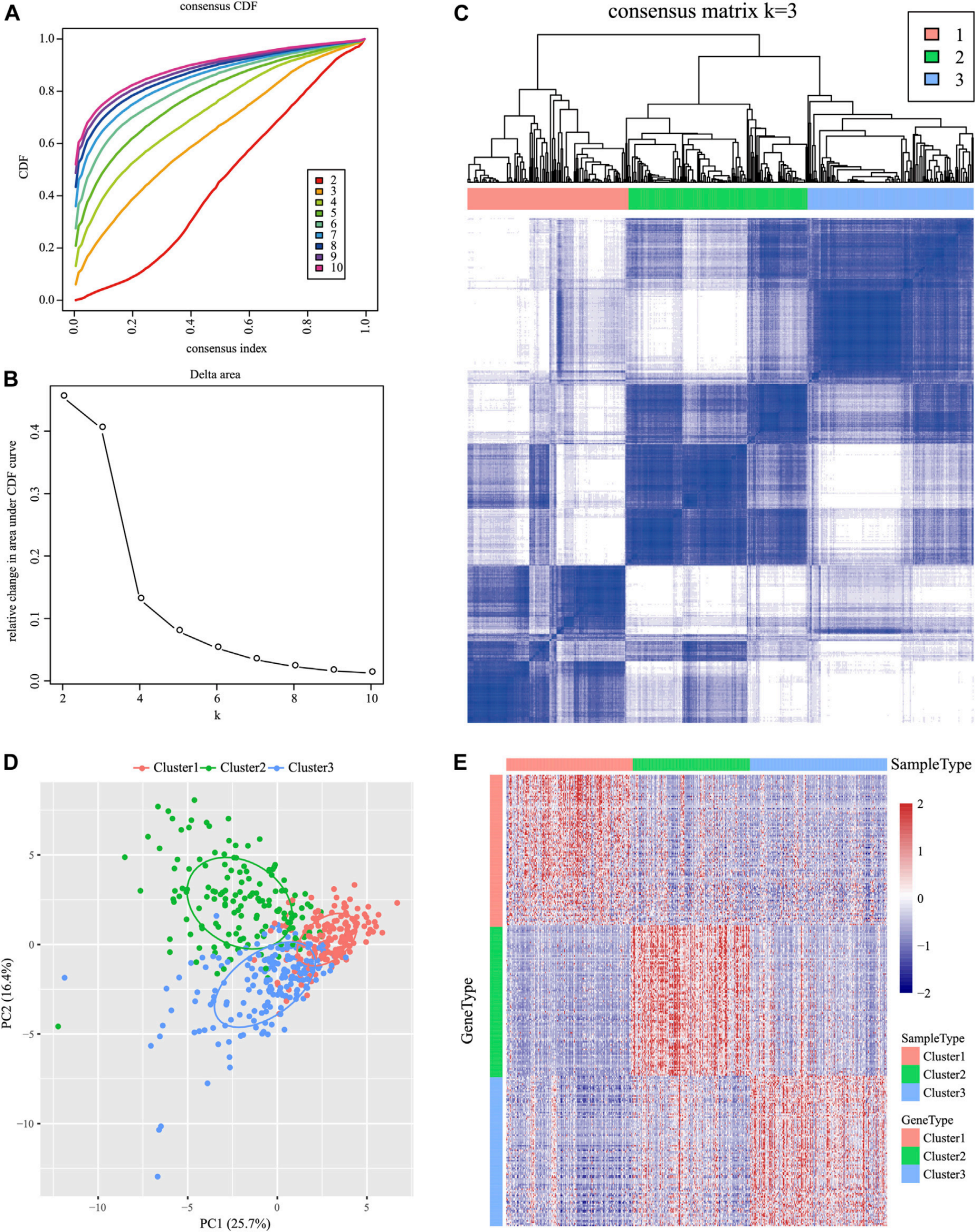
Identification of immune-associated subtypes of HNSCC in the TCGA cohort. **(A)** Cumulative distribution function (CDF) curves of consensus scores based on different subtype numbers (k = 2–10) and the corresponding colors are represented. **(B)** CDF Delta area curve of all samples when k = 3. **(C)** Consensus score matrix of HNSCC samples when k = 3 (1 = C1, 2 = C2, and 3 = C3). **(D)** Principal component analysis (PCA) of the gene expression profile of the upregulated genes. **(E)** Gene expression heatmap analysis of the top 100 genes that were significantly upregulated in each subtype.

For the 939 IRGs, their gene expression patterns were utilized for examining HNSCC subtypes based on the TCGA cohort. To be specific, all cancer samples were classified as k (k = 2, 3, 4, 5, 6, 7, 8, 9, and 10) distinct subtypes by the use of ConsensusClusterPlus. In line with CDF curves regarding consensus score, k = 3 resulted in the best division (Figures 1A, B). In addition, SigClust analysis revealed significant consensus clusters (k = 3) upon pairwise comparisons (Figure 1B). Moreover, the difference in the distribution of expression between C1 and C2 subtypes was not significant, but those between C1 and C3 subtypes, and between C2 and C3 subtypes, were significant (*p* < 0.05). Therefore, those three sample clusters were isolated, and then the 500 HNSCC cancer samples collected based on the TCGA cohort were divided into three molecular subtypes according to the entire expression profiles of immune genes (Figure 1C).

### Characterizations of the Three Subtypes Within the TIME

The Kolmogorov–Smirnov test was utilized to analyze those upregulated IRGs for every molecular subtype relative to others (FDR <0.05). Of those 939 IRGs, 95 for the C1 subtype, 437 for the C2 subtype, and 333 for the C3 subtype remarkably increased in their expression. Thereafter, those 100 most significantly upregulated genes were screened in every subtype to perform PCA (Figure 1D). As revealed by PCA, all the aforementioned genes might be divided into three subtypes. To better identify the expression profiles of genes in every subtype, the 100 most significant genes screened from every subtype were analyzed by the heat map (Figure 1E), which revealed different expression profiles for those upregulated genes selected from every subtype.

### Subtype Clinical Features

**FIGURE 2:**
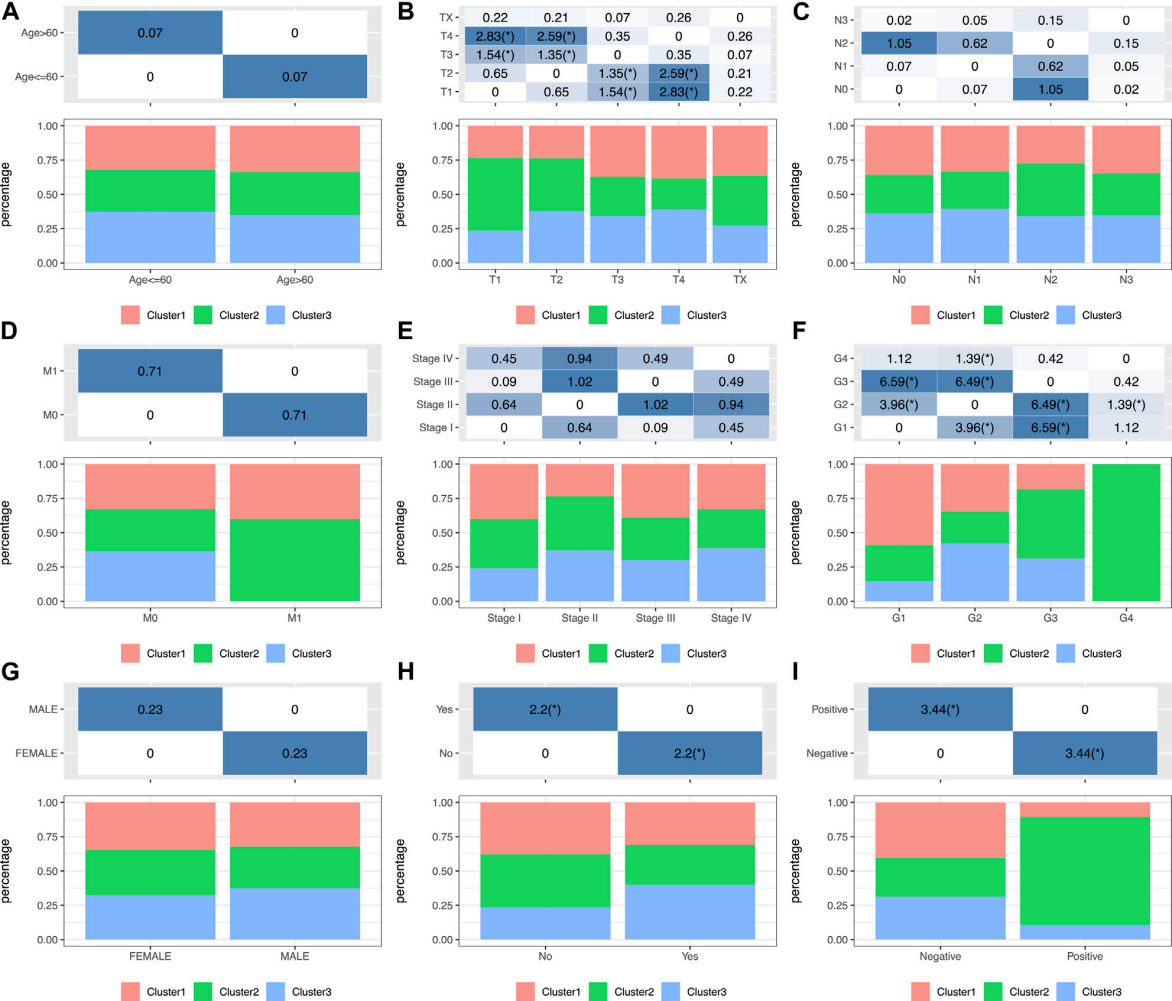
Factor analysis of three HNSCC subtypes based on clinical characteristics: age **(A)**, T staging ratio **(B)**, N staging ratio **(C)**, M staging ratio **(D)**, stage ratio **(E)**, histological grade ratio **(F)**, gender ratio **(G)**, smoking history **(H)** and HPV status **(I)** distribution in the three HNSCC subtypes.

To investigate the clinical significance of the TIME, this study analyzed numerous clinical features such as gender, age, grade, stage, tumor, node, metastasis (TNM) classification, smoking status, and HPV status among those three subtypes. The results showed that a different distribution in T stage was found among the three subtypes (Figure 2B) and that patients with T1 were significantly higher in C2 than those in the other two groups. Moreover, the significance of grade among those three subtypes (Figure 2F; Supplementary Table S2) revealed remarkably increased proportions of grade 1 for the C1 subtype and grade 3 for the C2 subtype (*p* < 0.001 upon log-rank test). In addition, this study estimated the distributions of smoking status among three subtypes (Figure 2H), which suggested markedly increased proportions of non-smokers for the C2 subtype and smokers for the C3 subtype (*p* < 0.01). Moreover, the study showed that more HPV-positive tumor patients were found in C2 than in the other two groups (Figure 2I). In addition, differences in the distributions of gender, age (threshold of 60), stage, N, and M classification were not significant (*p* > 0.05) (Figures 2A,C–E,G; Supplementary Table S2).

### HNSCC Immunogenicity

**FIGURE 3:**
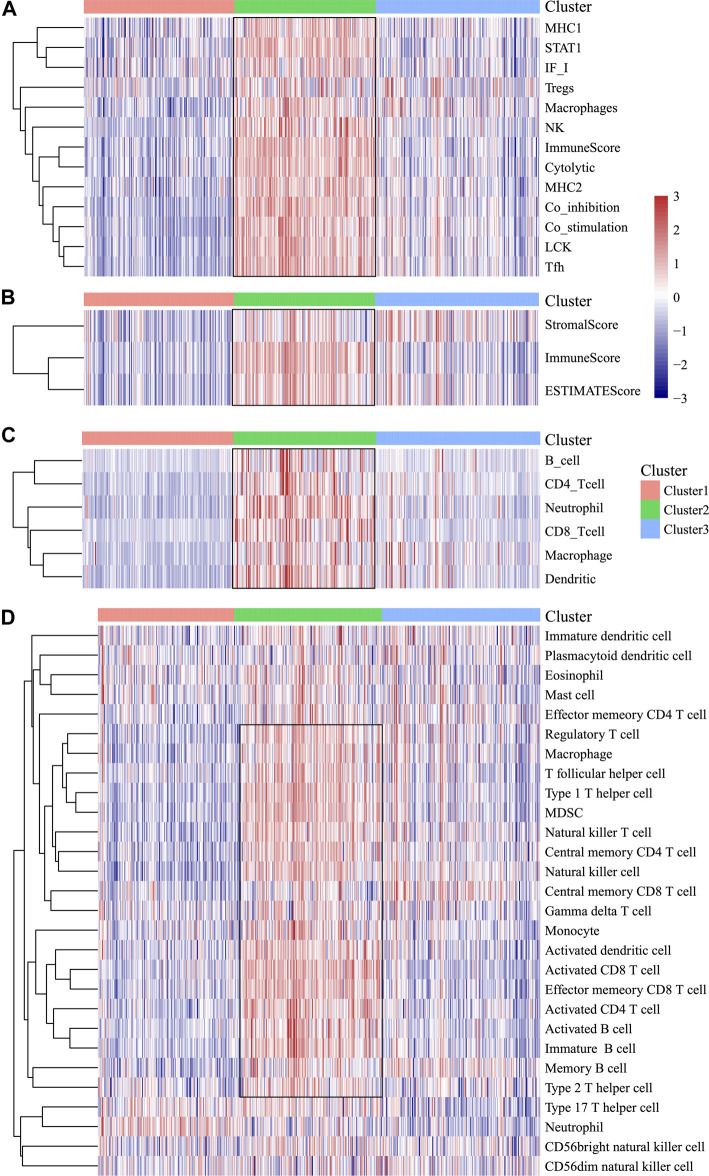
Immune profiles of the three HNSCC subtypes in the TCGA cohort. **(A)** Scores of 13 types of immune metagenes in the three HNSCC subtypes are displayed. **(B)** Tumor stroma scores, the immune scores, and the ESTIMATE scores of the three HNSCC subtypes are displayed. **(C)** Scores of six types of immune infiltrating cells among the three HNSCC subtypes are shown. **(D)** Scores of 28 groups of immune related pathways across three subtypes are shown.

**FIGURE 4:**
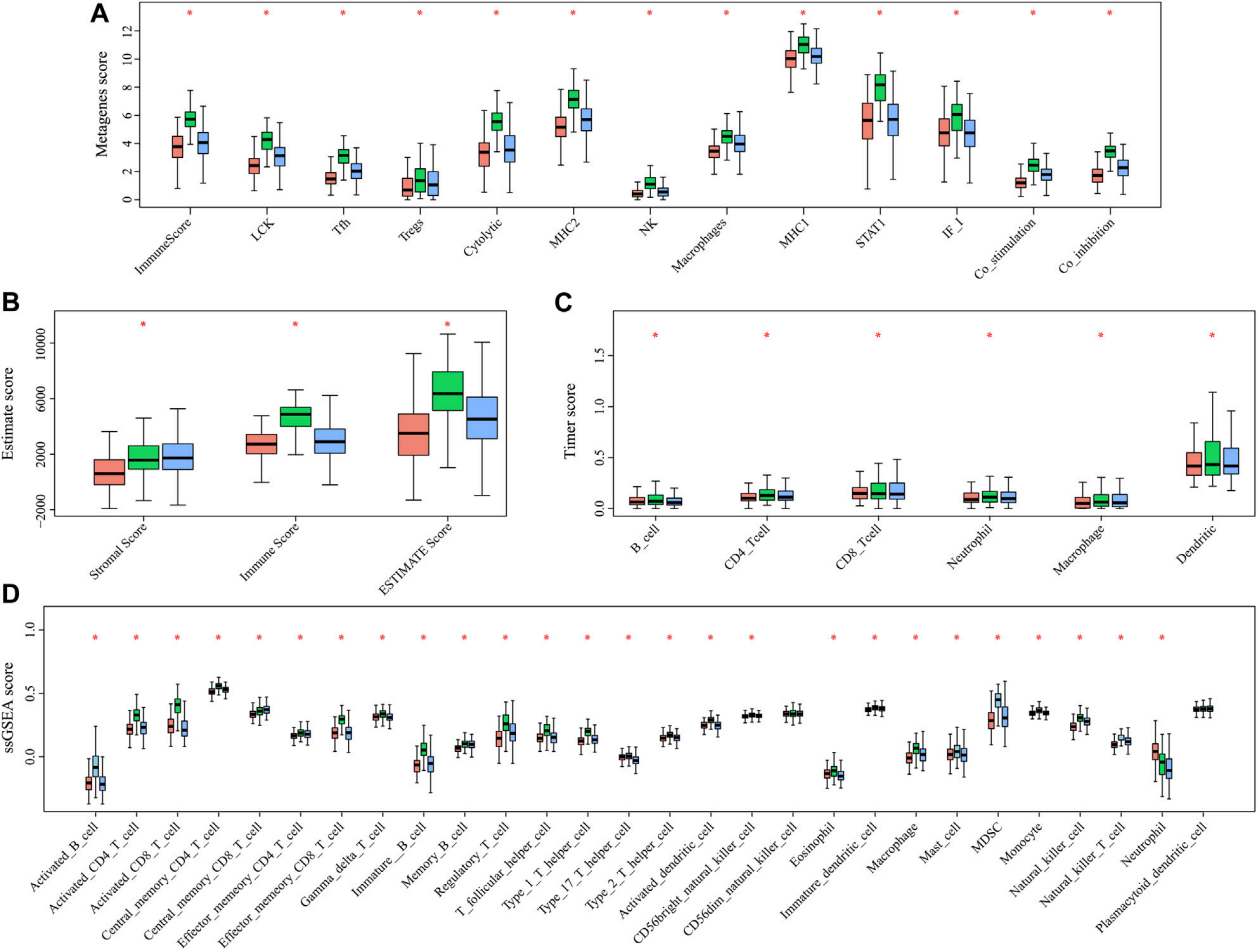
Multiple immune scores of the three HNSCC subtypes in the TCGA cohort. **(A)** Expression scores of 13 types of metagenes of the three HNSCC subtypes. **(B)** Boxplots indicating the tumor stroma scores, the immune scores, and the ESTIMATE scores of the three HNSCC subtypes. **(C)** Scores of six types of immune infiltrating cells of the three HNSCC subtypes. **(D)** Scores of 28 immune-related pathways of the three HNSCC subtypes.

In order to analyze the relationship between three subtypes and immunity, the scores for 13 types of immune metagenes, components of tumor immunity, and six types of infiltrating immunocytes, together with 28 immune-associated pathways, were determined. As a result, a majority of metagenes showed overexpression within the C2 subtype, whereas a downregulation was seen within the C1 and C3 subtypes (Figures 3A, 4A). In addition, the C2 subtype had markedly increased immune and stromal scores, but these characteristics were much lower in other subtypes (Figures 3B, 4B). In addition, scores for those six types of infiltrating immunocytes were remarkably increased for the C2 subtype (Figures 3C, 4C). Among those 28 immune-associated pathways, the C2 subtype had evidently increased scores relative to others, with the only exception of neutrophils- and plasmacytoid dendritic cell (DC)–associated immune pathways (Figures 3D, 4D). Collectively, the C2 subtype had the most upregulated immune signature of not only the other subtypes but also normal tissue, which indicates its superior immune profile.

### Clinical Relevance of Three Molecular Subtypes in Prognosis Prediction

**FIGURE 5:**
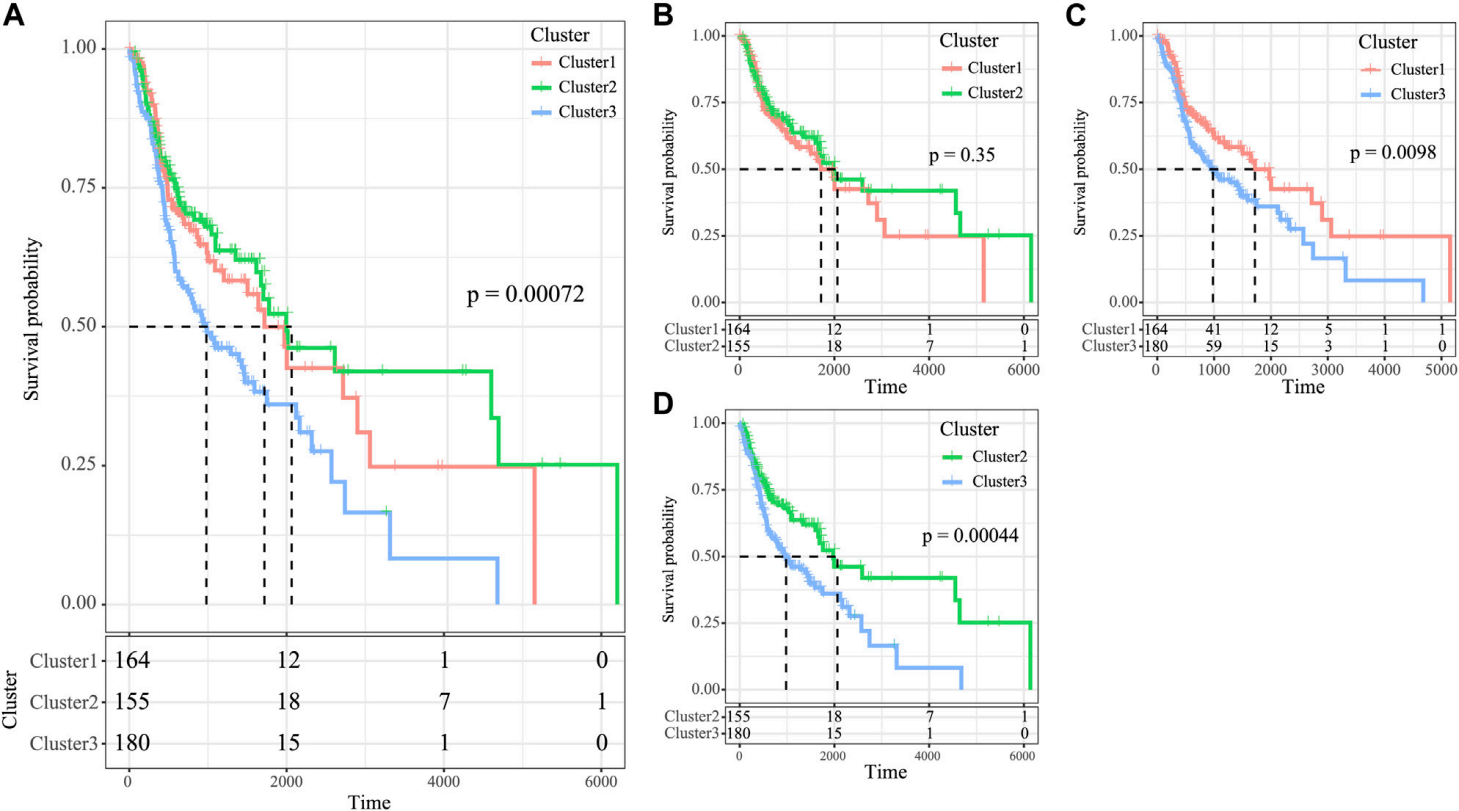
Survival analysis of the three HNSCC subtypes. **(A)** KM curve of OS prognosis of three subtypes. **(B)** Prognosis difference KM curve of C1 and C2. **(C)** Prognosis difference KM curve of C1 and C3 **(D)** Prognosis difference KM curve of C2 and C3.

The high tumor relapse and progression rates have resulted in a dismal prognosis for HNSCC. In line with differently expressed immune profiles, the relationship between HNSCC prognosis and those three subtypes was explored. The K–M curves revealed that differences in overall survival (OS) were significant among three subtypes of HNSCC cases in the TCGA cohort (*p* = 0.0072, Figure 5A). Among them, the C3 subtype showed the poorest prognosis, whereas the C2 subtype displayed more favorable prognosis (Figure 5A). To explore the associations between patient prognosis and immune signature, this study compared OS among the three subtypes. The results suggested that the C2 subtype, the immune-enhanced subtype that showed greater immune scores, exhibited superior prognosis (Figure 5B–D, C1 vs. C3, *p* = 0.0098, C2 vs. C3, *p* = 0.00044, and C2 vs. C1, *p* = 0.35).

### Gene Mutation Frequencies Among Three Molecular Subtypes

**FIGURE 6:**
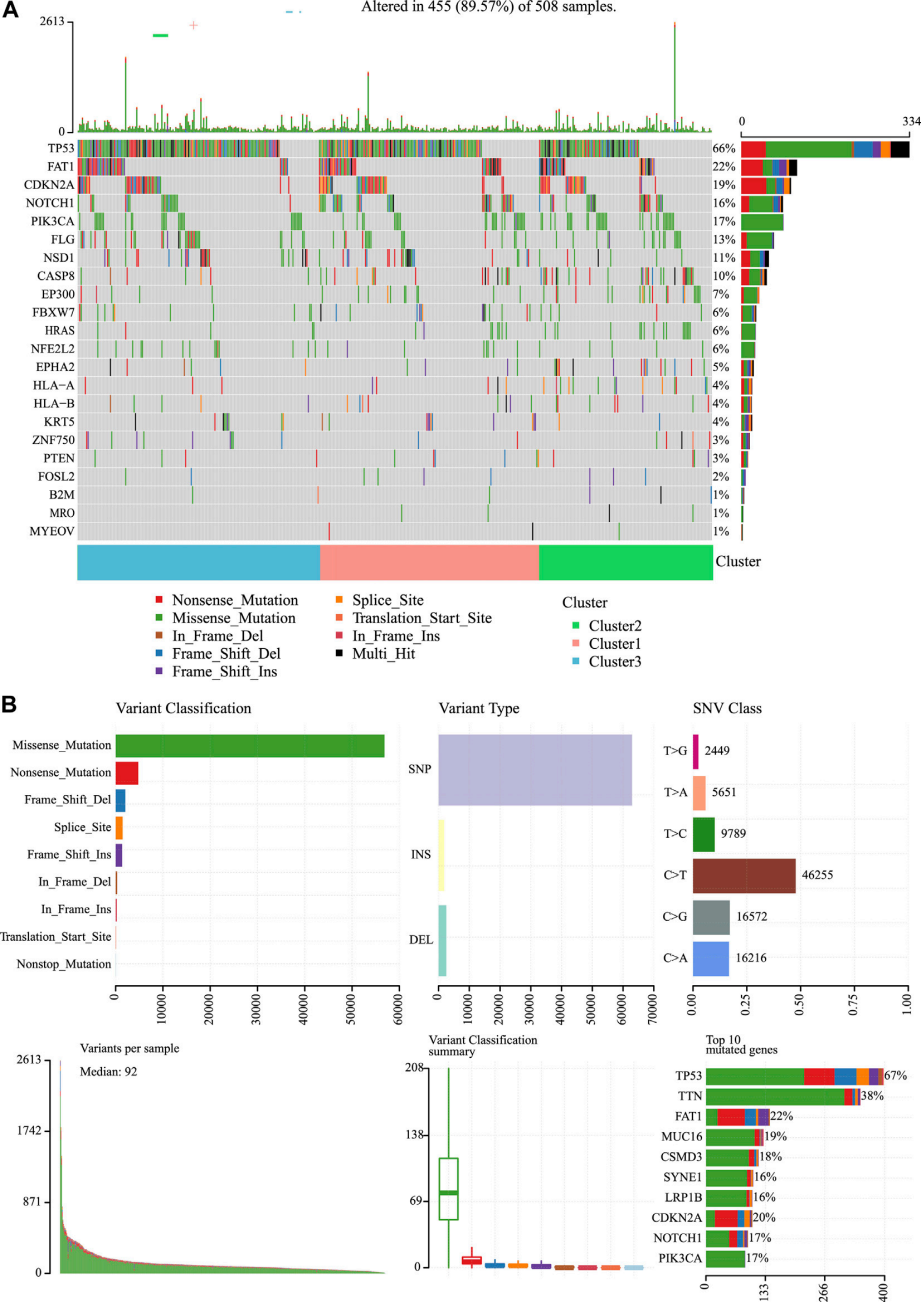
Mutation analysis for the three subtypes of HNSCC. **(A)** Somatic landscape of HNSCC cohort. **(B)** Distribution of the gene with a higher mutation frequency in different mutation types and the change of nucleotide sites.

We then analyzed the mutation genes for three subtypes to gain further insight into some biomarkers related to HNSCC immunogenicity. First of all, Mutsig2 (Pickering et al., 2014) was utilized to identify genes with significant mutations at the threshold of FDR<0.05. A total of 32 genes with significant mutation frequencies were obtained. Figure 6 exhibits the distributions of silent mutations, missense mutations, framework insertion or deletion, framework shift, nonsense mutation, splice sites, and other non-synonymous mutations of the top 22 genes with significant mutation frequencies in the three subtypes (Figure 6A). As revealed by our observations, the C2 subtype had a markedly decreased proportion of mutations among the three most significant genes (CDKN2A, TP53, and FAT1) compared with other subtypes. Furthermore, it was discovered that over half of these mutation sites were C > T. The SNP mutation distributions of all samples are displayed in Figures 2C, 6B).

### Analysis of the Gene Co-Expression Network

**FIGURE 7:**
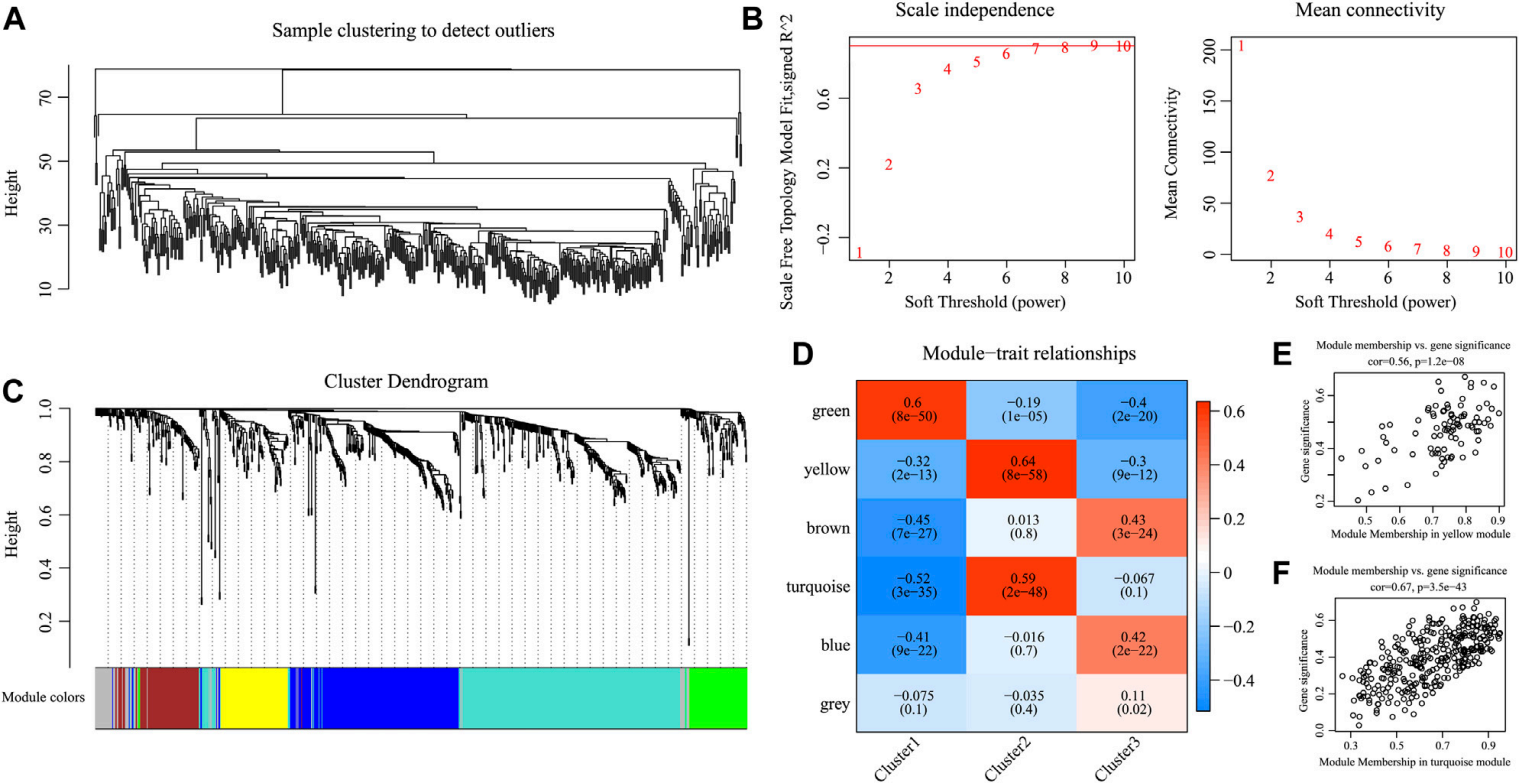
Weighted gene co-expression network analysis (WGCNA) of differentially expressed immune-related genes in the three HNSCC subtypes in the TCGA cohort. **(A, B)** Analysis of network topology for various soft thresholding powers. **(C)** Hierarchical cluster tree displaying seven modules of co-expressed genes. **(D)** Heatmap showing the correlation between feature vectors of six modules and three HNSCC subtypes. **(E,F)** Gene significance (*y*-axis) vs. module membership (*x*-axis) plotted for yellow module **(E)** and turquoise module **(F)** in the TCGA dataset.

For better examination of candidate markers related to the TIME in HNSCC, expression data for 865 IRGs of three subtypes were collected. Then, Pearson’s correlation coefficient was utilized to calculate the distance among diverse transcripts. For the sake of constructing a scale-free network, this study set the β value of 6 (Figures 7A, B). According to TOM, the genes were clustered by average-linkage hierarchical clustering in accordance with hybrid dynamic cut tree criteria, with a minimal gene number of 30/module. When the gene module was determined by the dynamic shear approach, this study calculated the eigengenes in every module and conducted corresponding clustering analysis. Finally, six modules covering 865 IRGs with differential expressions were discovered by WGCNA (Figure 7C). Genes in the gray module could not be clustered in the other modules. Supplementary Table S3 shows the transcript statistics for every module. Altogether, 805 transcripts were classified into five co-expression modules. Subsequently, this study determined the associations of eigengenes within the six modules with those three subtypes (Figures 7D–F). As a result, the turquoise and yellow modules showed a positive correlation with the C2 subtypes but a negative correlation with the C1 and C3 subtypes. In addition, the green module showed a positive correlation with the C1 subtype, but negative correlation with the C2 and C3 subtypes. The blue and brown modules displayed a positive correlation with the C3 subtype but a negative correlation with the C1 subtype.

**FIGURE 8:**
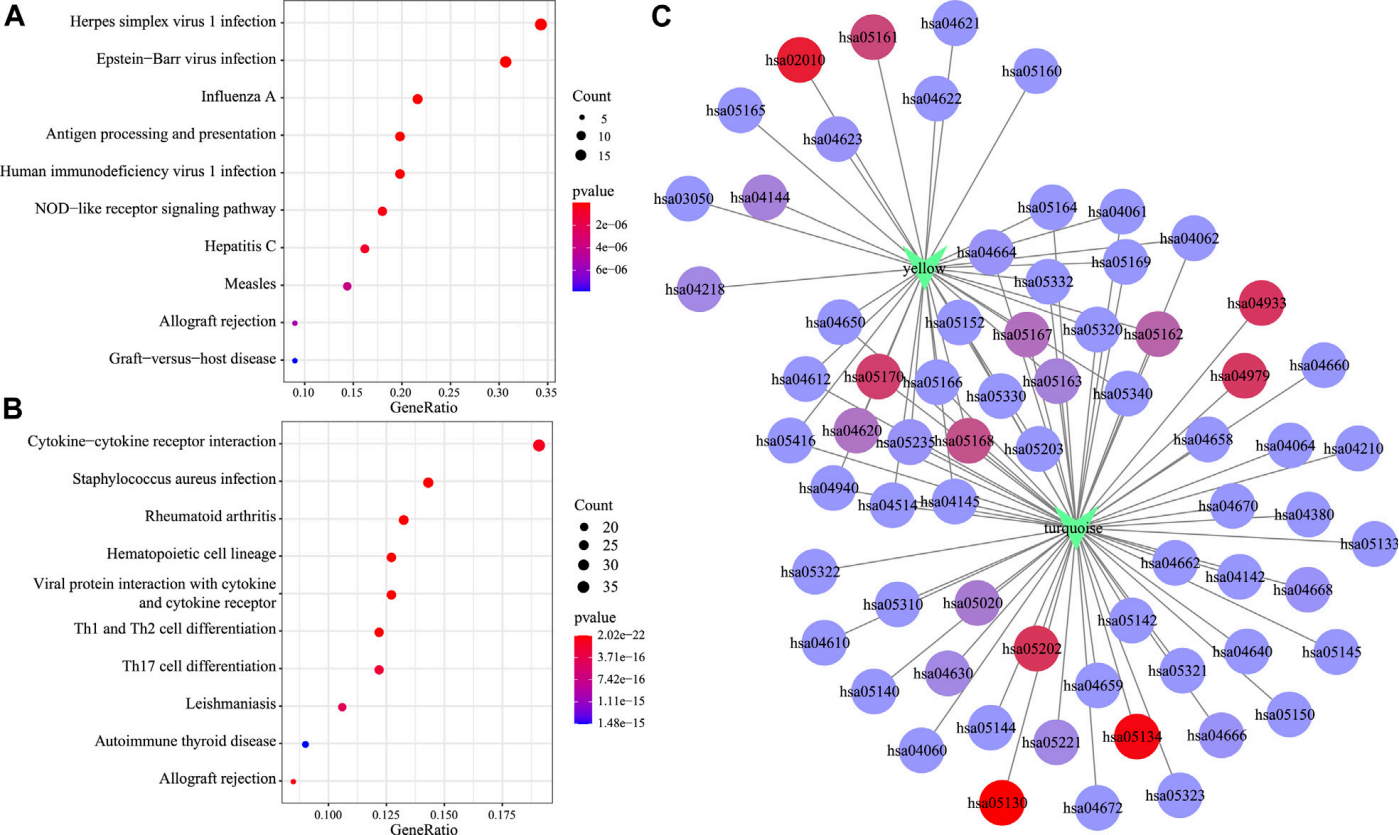
Functional analysis of gene modules significantly related to subtypes C2. KEGG enrichment analysis results of genes in the yellow module **(A)** and the turquoise module **(B)**. Intersection network of enrichment pathways between the two modules **(C)**.

Moreover, KEGG pathway enrichment analysis was conducted to illustrate the biological functions of genes in the turquoise and yellow modules that showed a positive correlation with the C2 subtype. The yellow module was primarily enriched into 33 pathways (the top 10 are displayed in Figure 8A), including antigen processing and presentation, EB virus, and herpes simplex virus type 1 infection. At the same time, the turquoise module was primarily enriched into 57 pathways (the top 10 are displayed in Figure 8B), including certain immune-associated pathways such as an interaction between cytokine and cytokine receptor and T-cell differentiation. Afterward, this study visualized the network of relationships regarding those enriched pathways within the two modules. According to Figure 8C, the turquoise and yellow modules were mainly enriched into 22 common pathways (Supplementary Table S4), which suggested that genes within these two modules had parallel regulatory processes.

### External Validation for Three Subtypes

Based on the abovementioned co-expression gene modules (turquoise and yellow), altogether 314 featured genes that had a correlation coefficient of > 0.5 were acquired and their expression profiles were then collected into the training set and samples were classified by the SVM, which achieves an accuracy rate of 100% in classification. For better validation of those three subtypes, this study classified 270 samples by the use of the SVM. Among them, 89 samples were classified into the C1 subtype, 102 into the C2 subtype, and 79 into the C3 subtype.

**FIGURE 9:**
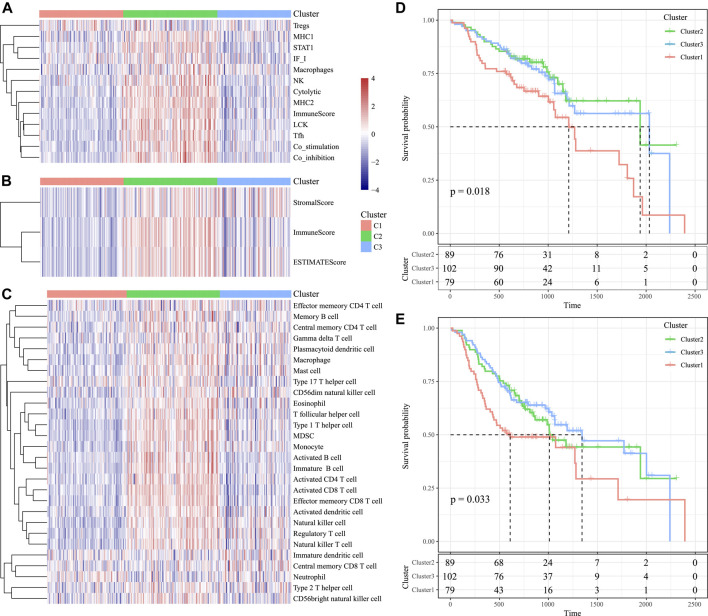
Immune profiles and survival analysis of three HNSCC subtypes in the validation set. **(A)** Scores of 13 types of immune metagenes in the three HNSCC subtypes are displayed. **(B)** Tumor stroma scores, the immune scores, and the ESTIMATE scores in the three HNSCC subtypes are displayed. **(C)** Scores of 28 immune-related pathways across the three HNSCC subtypes are shown. **(D)** KM curve of OS prognosis of three subtypes. **(E)** KM curve of PFS prognosis of three subtypes.

According to our results, for those 13 immune metagenes, their expression distributions within three subtypes were examined (Figure 9A). A majority of metagenes showed high expression within the C2 subtype. In addition, the C2 subtype had an evidently increased immune score and stromal scores relative to those in other subtypes (Figure 9B). Scores of the immune-associated pathways among the samples were further analyzed, as presented in Figure 9C. It was discovered that the C2 subtype had remarkably increased scores relative to those of other subtypes, as verified by results from the training set. Last, differences in OS and progression-free survival (PFS) for HNSCC cases were significant among three subtypes (*p* = 0.018 for OS, *p* = 0.033 for PFS, Figures 9D, E). Cases in the C3 subtype showed the poorest prognosis, whereas those in C2 subtype displayed the most favorable prognosis, as verified by the abovementioned results from the training set. The abovementioned findings indicated that the presence of an immune-enhanced subtype within HNSCC showed significant differences compared with the other two subtypes.

## Discussion

HNSCC represents a highly aggressive form of cancer, and it ranks among the top causes of cancer-related deaths. HNSCC genesis and progression is closely related to the infiltration and modification of immune cells and immune escape in the tumor microenvironment (Miyauchi et al., 2019). This work comprehensively analyzed the three subtypes of HNSCC microenvironment based on the global immune genes and explored the corresponding clinical relevance through the use of TCGA-derived data.

With an increased immune profile, the C2 subtype had elevated immunocyte infiltration scores, and they exhibited positive correlation with signatures of multiple types of immune-related cells and pathways when compared with C3. The abundant stroma in the tumor microenvironment could be conducive to the growth and metastasis of tumor cells, which also influences the antitumor immune effect (Hamidi and Ivaska, 2018). Therefore, the C1 subtype may be a stroma-deficient type, which may be more efficient for the immune response than the C3 subtype. Therefore, the immune-enhanced subtype might co-exist with the relative immune-decreased subtype within the TIME of HNSCC, and there were significant differences in the expression profiles of metagenes, immune infiltration scores, immune component scores, as well as immune-associated pathways (Veigas et al., 2021).

For HNSCC cases, their prognostic outcome showed a positive correlation with the increased expression of immune-related cells and markers, such as macrophages and NK cell–associated molecules (Qian and Pollard, 2010; Bisheshar et al., 2020). M2 macrophage signatures are associated with favorable prognosis in HNSCC (Chen et al., 2019; Bouaoud et al., 2021). In addition, OX40 + plasmacytoid dendritic cells were enriched in the TME of HNSCC and generated specific CD8^+^ T cell responses to inhibit tumor growth (Poropatich et al., 2020). The STAT3-VSIR axis is an immune microenvironment marker that works by decreasing CD4 helper T-cell activity and is thus associated with poorer survival (Xu et al., 2020). Targeting CD276 can also eliminate HNSCC stem cells in a CD8^+^ T cell–dependent manner (Wang et al., 2021).

Our study further investigated the relationship between immune types and pathological types, clinical stages, and smoking. The recurrent tumors of HNSCC with poor therapeutic response and detrimental prognosis have an immunosuppressive TIME (Watermann et al., 2021). Active smoking in HNSCC may have a remarkable immunosuppressive effect. Suppression of T-cell chemotaxis may be a key factor in the relationship between smoking and TIME (de la Iglesia et al., 2020). In addition, T1 patients who have a good prognosis were more in immune-enhanced patients than in other pathological types that are consistent with K–M survival analysis.

We identified the candidate targets and related pathways for those three subtypes within the TIME through WGCNA. To be specific, C2 was mainly enriched into the virus infection– and immune-associated pathways. These results indicate that genes from turquoise and yellow modules potentially have a parallel effect on the TIME of HNSCC, which were also related to 22 common pathways in the KEGG database. Viruses such as HPV and EB are factors that promote HNSCC. Previous research indicated that HPV-positive HNSSCs showed the highest levels of immune cell infiltration compared to other HNSSC types (Mandal et al., 2016). Single-cell RNA-seq analysis showed that helper CD4^+^ T cells and B cells in HNSCC of HPV-positive and HPV- negative patients are relatively divergent (Cillo et al., 2020). Tumor infiltrating B cells increased in HPV-positive HNSCC patients compared to HPV-negative patients, and their specific phenotype and localization contributed to overall survival in the TIME (Ruffin et al., 2021). Virus-related HNSCC may show a higher response to immunotherapy in clinical trials (Perri et al., 2020). Notably, innate immunity is vital for the modulation of HNSCC genesis and progression (McCormick et al., 2016). Moreover, HNSCC progression is suggested to induce the adaptive immune response (Wondergem et al., 2020). Thus, it is important to classify related molecular mechanisms on the basis of the TIME in HNSCC, which may contribute to identifying new chemopreventive targets for the treatment of HNSCC.

Mutations in chromatin modifier genes are an important mechanism of oncogenesis. The mutations in TP53, CDKN2A, and FAT1 genes reported in prior works are found to be tightly associated with HNSCC development (Coombes et al., 2003; Eriksen et al., 2005). The TP53 mutation is a central site in many cancers and is commonly detected in HNSCC. TP53 is frequently linked with poorer survival and a more aggressive form of cancer (Vousden and Prives, 2005). Otherwise, there is a similar association between TP53 and other high-rate genes in HNSCC. For example, TP53 and CDKN2A have co-mutations in throat squamous cell carcinoma and oral squamous cancer (Todorova et al., 2015). CDKN2A is a basic gene within the cell cycle, also called p16, which is directly involved in the regulation of the cell cycle and negatively regulates cell proliferation and division. Once CDKN2A deletion or mutation occurs, it will lead to malignant cell proliferation and participate in tumor formation. FAT1 encodes a protocaladherin, which is very frequently mutated in many human cancers, especially squamous cell carcinomas (SCCs) (Dotto and Rustgi, 2016). Recently, it has been confirmed that the loss of FAT1 function promotes tumorigenesis, development, invasion, and metastasis by inducing mixed epithelial-to-mesenchymal transition (EMT) status in human squamous cell carcinoma (Pastushenko et al., 2021). These three types of gene mutations are less likely to occur in the immune-enhanced subtype, indicating a better survival rate.

Certain limitations should be noted in this work. First of all, further studies should enroll a larger number of clinical features and treatment characteristics for HNSCC patients to carry out subgroup analysis so as to explore the influencing factors and their impact on the phenotypes of the HNSCC microenvironment. Second, only the NCI cohort was adopted in external validation, which may lead to one-sided results, together with a high false-positive rate. Third, the differential expression of IRGs should be further examined in the three subtypes to reveal the immune escape mechanisms of HNSCC and to provide a molecular and pathological foundation for individualized targeted immunotherapy.

Taken together, the immune microenvironment phenotypes in HNSCC can be divided into three molecular subtypes according to the possible mechanisms of immune escape within HNSCC. These three molecular subtypes have different immune features, mutations of oncogenes, and prognosis for patients. In addition, some functional pathways can trigger microenvironment phenotype formation. The abovementioned findings further confirm that the HNSCC immune heterogeneity could support and predict the prognosis of HNSCC patients. The concept can also shed more light on the development of a new personalized treatment of HNSCC through the immune microenvironment to monitor patients.

## Data Availability

The original contributions presented in the study are included in the article/Supplementary Material, further inquiries can be directed to the corresponding author.
