# Supplementary material for: Systemic Analysis on the Features of Immune Microenvironment Related to Prognostic Signature in Head and Neck Squamous Cell Carcinoma
Source: Front Genet. 2022 May 11;13:860712. doi: 10.3389/fgene.2022.860712 (PMC9130752; doi:10.3389/fgene.2022.860712)
Supplement: Supplementary file 1 [file Table1.DOCX]

**Supplemental Table 1. Clinical information statistics of two data sets**

| **Characteristic** | | **TCGA datasets(n=500)** | | **GSE65858(n=270)** |
| --- | --- | --- | --- | --- |
| **Age(years)** | <=60 | 244（48.8%） | 153（56.7%） | |
|  | >60 | 256（51.2%） | 117（43.3%） | |
| **Survival Status** | Living | 283（56.6%） | 176（65.2%） | |
|  | Dead | 217（43.4%） | 94（34.8%） | |
| **Gender** | female | 133（26.6%） | 47（17.4%） | |
|  | male | 367（73.4%） | 223（82.6%） | |
| **Grade** | G 1 | 61（12.2%） | / | |
|  | G 2 | 299（59.8%） | / | |
|  | G 3 | 119（23.8%） | / | |
|  | G 4 | 2（0.4%） | / | |
|  | GX | 19（3.8%） | / | |
| **pathologic_T** | T 1 | 34（6.8%） | 35（13.0%） | |
|  | T 2 | 143（28.6%） | 80（29.6%） | |
|  | T 3 | 132（26.4%） | 58（21.5%） | |
|  | T 4 | 191（38.2%） | 97（35.9%） | |
| **pathologic_N** | N 0 | 241（48.2%） | 94（34.8%） | |
|  | N 1 | 81（16.2%） | 32（11.9%） | |
|  | N 2 | 152（30.4%） | 132（48.9%） | |
|  | N 3 | 26（5.2%） | 12（4.4%） | |
| **pathologic_M** | M 0 | 475（95.0%） | 263（97.4%） | |
|  | M 1 | 5（1.0%） | 7（2.6%） | |
|  | MX | 20（4.0%） | / | |
| **Tumor Stage** | Stage Ⅰ | 25（5.0%） | 18（6.7%） | |
|  | Stage Ⅱ | 81（16.2%） | 37（13.7%） | |
|  | Stage Ⅲ | 90（18.0%） | 37（13.7%） | |
|  | Stage Ⅳ | 304（60.8%） | 178（65.9%） | |
| **HPV** | HPV Negative | 7（1.6%） | 196（72.6%） | |
|  | HPV Positive | 30（6.9%） | 73（28.1%） | |
|  | Unknown | 398（91.5%） | 1（0.3%） | |
| **Smoking** | No | 111（22.7%） | 48（17.8%） | |
|  | Yes | 379（77.3%） | 222（82.2%） | |

**Supplemental Table 2: Relationship between three subtypes and clinical characteristics**

| **Characteristic** | | **Cluster 1(n=164)** | **Cluster 2(n=156)** | **Cluster 3(n=180)** | | **p value** |
| --- | --- | --- | --- | --- | --- | --- |
| **Age(years)** | <=60 | 78(47.6%) | 75(48.1%) | 91(50.6%) | 0.837 | |
|  | >60 | 86(52.4%) | 81(51.9%) | 89(49.4%) |  |  |
| **Survival Status** | Living | 103(62.8%) | 99(63.5%) | 81(45.0%) | 0.00044 | |
|  | Dead | 61(37.2%) | 57(36.5%) | 99(55.0%) |  |  |
| **Gender** | female | 46(28.0%) | 44(28.2%) | 43(23.9%) | 0.588 | |
|  | male | 118(72.0%) | 112(71.8%) | 137(76.1%) |  |  |
| **Grade** | G 1 | 36(22.0%) | 16(10.2%) | 9(5.0%) | 2.08E-10 | |
|  | G 2 | 106(64.6%) | 75(48.1%) | 132(73.3%) |  |  |
|  | G 3 | 22(13.4%) | 63(40.4%) | 38(21.1%) |  |  |
|  | G 4 | 0(0.0%) | 2(1.3%) | 1(0.6%) |  |  |
| **pathologic_T** | T 1 | 9(5.5%) | 18(11.5%) | 8(4.4%) | 0.0018 | |
|  | T 2 | 35(21.3%) | 57(36.6%) | 55(30.6%) |  |  |
|  | T 3 | 50(30.5%) | 39(25.0%) | 46(25.6%) |  |  |
|  | T 4 | 70(42.7%) | 42(26.9%) | 71(39.4%) |  |  |
| **pathologic_N** | N 0 | 86(52.4%) | 68(43.6%) | 87(48.3%) | 0.388 | |
|  | N 1 | 27(16.5%) | 22(14.1%) | 32(17.8%) |  |  |
|  | N 2 | 42(25.6%) | 58(37.2%) | 52(28.9%) |  |  |
|  | N 3 | 9(5.5%) | 8(5.1%) | 9(5.0%) |  |  |
| **pathologic_M** | M 0 | 162(98.8%) | 152(92.7%) | 173(100.0%) | 0.1956 | |
|  | M 1 | 2(0.2%) | 4(0.3%) | 0(0.0%) |  |  |
| **Tumor Stage** | Stage Ⅰ | 10(6.1%) | 9(5.8%) | 6(3.3%) | 0.194 | |
|  | Stage Ⅱ | 19(11.6%) | 32(20.5%) | 30(16.7%) |  |  |
|  | Stage Ⅲ | 35(21.3%) | 28(17.9%) | 27(15.0%) |  |  |
|  | Stage Ⅳ | 100(61.0%) | 87(55.8%) | 117(65.0%) |  |  |
| **HPV** | HPV Negative | 26(92.9%) | 18(54.5%) | 20(90.9%) | 0.000366 | |
|  | HPV Positive | 2(7.1%) | 15(45.5%) | 2(9.1%) |  |  |
| **Smoking** | No | 42(26.4%) | 43(27.6%) | 26(14.7%) | 0.0063 | |
|  | Yes | 117(73.6%) | 113(72.4%) | 151(85.3%) |  |  |

**Supplemental Table 3: Transcript data for six modules.**

| **Modules** | **Genes** |
| --- | --- |
| blue | 225 |
| brown | 90 |
| green | 80 |
| grey | 60 |
| turquoise | 321 |
| yellow | 89 |

**Supplemental Table 4: The Common Enriched Pathways within Turquoise and Yellow Modules**

| **ID** | **Pathway** |
| --- | --- |
| hsa04061 | Viral protein interaction with cytokine and cytokine receptor |
| hsa04062 | Chemokine signaling pathway |
| hsa04145 | Phagosome |
| hsa04514 | Cell adhesion molecules (CAMs) |
| hsa04612 | Antigen processing and presentation |
| hsa04620 | Toll-like receptor signaling pathway |
| hsa04650 | Natural killer cell mediated cytotoxicity |
| hsa04940 | Type I diabetes mellitus |
| hsa05162 | Measles |
| hsa05163 | Human cytomegalovirus infection |
| hsa05164 | Influenza A |
| hsa05166 | Human T-cell leukemia virus 1 infection |
| hsa05167 | Kaposi sarcoma-associated herpesvirus infection |
| hsa05168 | Herpes simplex virus 1 infection |
| hsa05169 | Epstein-Barr virus infection |
| hsa05170 | Human immunodeficiency virus 1 infection |
| hsa05235 | PD-L1 expression and PD-1 checkpoint pathway in cancer |
| hsa05320 | Autoimmune thyroid disease |
| hsa05330 | Allograft rejection |
| hsa05332 | Graft-versus-host disease |
| hsa05340 | Primary immunodeficiency |
| hsa05416 | Viral myocarditis |
